# Regional Heterogeneity of Cerebral Microvessels and Brain Susceptibility to Oxidative Stress

**DOI:** 10.1371/journal.pone.0144062

**Published:** 2015-12-02

**Authors:** Susan A. Austin, Anantha Vijay R. Santhanam, Livius V. d’Uscio, Zvonimir S. Katusic

**Affiliations:** Departments of Anesthesiology and Molecular Pharmacology & Experimental Therapeutics, Mayo Clinic College of Medicine, Rochester, Minnesota, United States of America; Nathan Kline Institute and New York University School of Medicine, UNITED STATES

## Abstract

The hippocampus is one of the earliest and most affected regions in Alzheimer’s disease (AD), followed by the cortex while the cerebellum is largely spared. Importantly, endothelial dysfunction is a common feature of cerebral blood vessels in AD. In this study, we sought to determine if regional heterogeneity of cerebral microvessels might help explain the susceptibility of the hippocampus and cortex as compared to the cerebellum. We isolated microvessels from wild type mice from the cerebellum, cortex, and hippocampus to characterize their vascular phenotype. Superoxide anion was significantly higher in microvessels isolated from the cortex and hippocampus as compared to the cerebellum. Importantly, protein levels of NADPH oxidase (NOX)-2 and NOX-4 were significantly higher in the cortical and hippocampal microvessels as compared to microvessels from the cerebellum. In addition, expression of manganese superoxide dismutase protein was significantly lower in microvessels from the cortex and hippocampus as compared to cerebellum while other antioxidant enzymes were unchanged. There was no difference in eNOS protein expression between the microvessels of the three brain regions; however, bioavailability of tetrahydrobiopterin (BH_4_), an essential cofactor for eNOS activity, was significantly reduced in microvessels from the hippocampus and cortex as compared to the cerebellum. Higher levels of superoxide and reduced tetrahydrobiopterin bioavailability may help explain the vulnerability of the hippocampus and cortical microvessels to oxidative stress and development of endothelial dysfunction.

## Introduction

It is believed that vascular dysfunction may play a role in Alzheimer’s disease (AD). Cardiovascular and cerebrovascular risk factors, such as midlife hypertension, hypercholesterolemia, diabetes mellitus, obesity, and a sedentary lifestyle as well as stroke are often associated with a higher incidence of AD [1,2]. Cerebrovascular disease is often found in AD [3,4,5]. Blood flow changes and other vascular abnormalities are a common feature of AD [6,7]. Iadecola et al (1999) and Niwa et al (2002) have described endothelial dysfunction as an early event in disease progression in AD transgenic mouse models [8,9,10]. Indeed, we have recently demonstrated in endothelial nitric oxide synthase (eNOS) deficient (eNOS^-/-^) mice, that loss of endothelial nitric oxide (NO) leads to AD-related changes in amyloid precursor protein (APP) and beta-amyloid (Aβ) levels in brain tissue, including the hippocampus [11]. Taken together, these studies suggest that the integrity of normal vascular function could be an important mechanism in protecting brain tissue from the pathological processes in AD.

The hippocampus is the earliest region affected by AD [12]. As the disease progresses, pathology spreads into the cortical regions of the brain while the cerebellum is largely spared. The spatial specific initiation and spreading of disease pathology within the brain is not well understood. We hypothesize that the heterogeneity of cerebral microvessels might help explain the susceptibility of brain regions to oxidative stress and other insults.

Vascular heterogeneity has been well described in the different vascular beds in the periphery (reviewed [13]). These differences may allow for tissue specific structural or functional roles of that particular vascular bed. However, a characterization of the differences within the cerebral circulation under normal physiological conditions has not been performed. Some groups have begun to characterize the gene and protein expression within the cerebrovasculature [14,15]. MacDonald et al (2010) examined the heterogeneity in gene expression of the endothelium of the blood brain barrier and Saubamea et al (2012) examined glycoprotein expression with the cerebral vasculature [14,15]. In this study, we examined microvessels from the cerebellum, a region that is largely spared in age-related cognitive decline and AD, and microvessels from the hippocampus and cortex, regions which are greatly affected. Our studies demonstrated important differences between microvessels isolated from the cerebellum and cortex and hippocampus in terms of levels of superoxide anion and bioavailable tetrahydrobiopterin (BH_4_), both important indicators of endothelial health and function. This suggests that the hippocampal and cortical microvessels may be more susceptible to oxidative stress and other insults.

## Materials and Methods

### Animals

Male wild type (C57BL6) mice were purchased from Jackson Laboratory (Bar Harbor, ME). Mice had free access to food and water. Mice were sacrificed by lethal dose of pentobarbital at 6–8 months old. All animal care and use were approved by Mayo institutional Animal Care and Use Committee.

### Tissue collection

Brains were carefully removed and immediately placed in ice cold modified Krebs-Ringer bicarbonate solution plus protease inhibitors. Large arteries, including the basilar and cerebral arteries, were removed. The cerebellum, cortex, and hippocampus were dissected out from two mice and pooled as one sample. Microvessels were isolated from the three regions.

### Microvessel isolation

Cerebral microvessels were isolated from the cerebellum, cortex, and hippocampus using the protocol previously described [16]. Individually, cerebellum, cortex, and hippocampal tissue were homogenized in ice cold PBS with Dounce homogenizer. Microvessels were isolated by layering over 15% Dextran/PBS solution and filtered using a 40 μm filter.

### Western blot

Microvessel tissue homogenates were lysed in ice cold Triton-X lysis buffer. Equal protein amounts were resolved by SDS-PAGE and transferred to nitrocellulose membranes. Blots were probed with anti-eNOS (BD Transduction Laboratories), catalase (Sigma, St. Louis, MO), cyclooxygenase (COX) -1 (Invitrogen, Camarillo, CA), inducible (i)NOS (BD Transduction Laboratories), manganese (Mn) superoxide dismutase (SOD), copper-zinc (CuZn) SOD, extracellular (EC) SOD (Enzo Life Sciences, Farmingdale, NY), neuronal (n)NOS (Cell Signaling, Billerica, MA), NADPH oxidase (NOX)-2 (Abcam, Cambridge, United Kingdom), NOX-4 (Novus Biologicals, Littleton, CO), or prostaglandin I2 synthase (PGI2S) (Cayman Chemical, Ann Arbor, MI) primary antibodies.

### Intracellular superoxide anion

Isolated microvessels were incubated with Kreb’s-Hepes with 50 μmol/L dihydroethidium (Molecular Probes, Eugene, OR) at 37° for 15 minutes. Microvessels were homogenized in methanol and intracellular superoxide anions were quantified using HPLC-based fluorescence and normalized using mg protein [17]. For some experiments, microvessels were incubated with 30 mmol/L N(Ω)-Nitro-L-arginine methyl ester (L-NAME), to inhibit eNOS prior to incubation with dihydroethidium.

### Biopterin levels

Microvessels were homogenized in ice cold extraction buffer (50 mmol/L Tris (pH 7.4, 1 mmol/L EDTA) with 1 mmol/L dithiothreitol. Samples were centrifuged and supernatants used for biopterin measurements. Tetrahydrobiopterin, BH_4_, and its oxidized product, 7,8-dihydobiopterin, BH_2_, were quantified using HPLC-based fluorescence and normalized per mg protein as previously described [17].

### cGMP levels

cGMP levels in microvessels were measured using a colorimetric immunoassay according to manufacturer’s instructions as previously described (Cell BioLabs, Inc. San Diego, CA) [11].

### Statistical analysis

Data are presented as mean ± SEM (n = number of mice). Data were analyzed using one-way ANOVA with Dunnett’s method post hoc comparison (comparing each group back to the cerebellum) using Sigma Plot12. A p value of <0.05 was considered statistically significant.

## Results

### Increased superoxide anion in cortical and hippocampal microvessels

**Fig 1:**
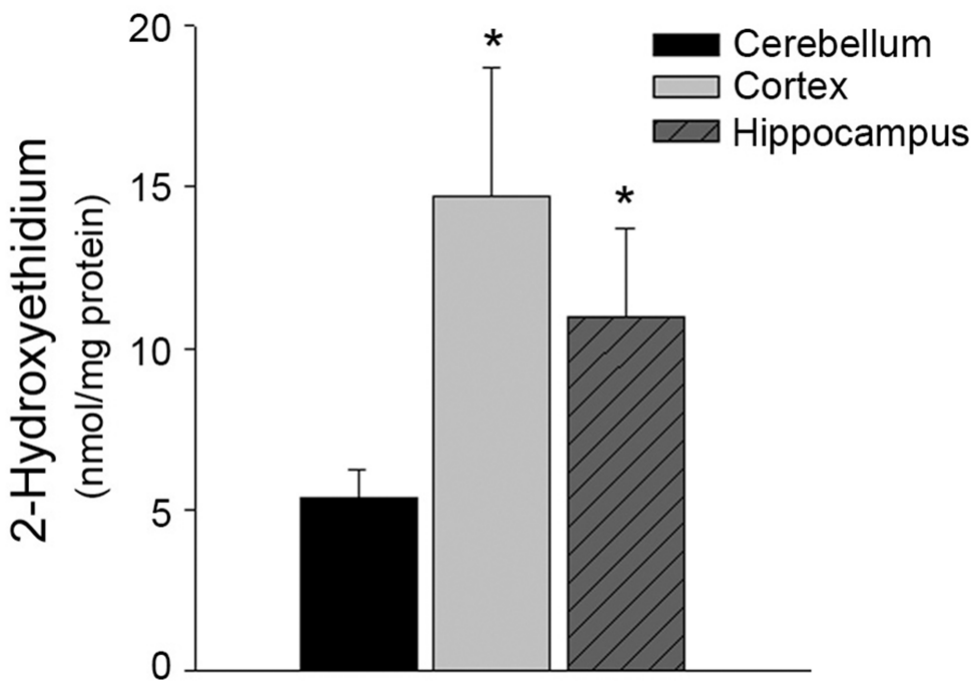
Intracellular superoxide anions are increased in the microvessels isolated from the cortex and hippocampus. Regional microvessels were isolated and incubated with 50 μmol/L dihydroethidium at 37° for 15 minutes. Microvessels were homogenized in methanol and intracellular superoxide anions quantified by HPLC-based fluorescence. Intracellular superoxide anions were normalized to mg protein of each sample (n = 12–13). Data are represented as mean ± SEM (P<0.05 as compared to cerebellum microvessels).

Intracellular superoxide anions were higher in microvessels isolated from the cortex and hippocampus as compared to the microvessels from the cerebellum (Fig 1; n = 12–13, P<0.05).

### NOX-2 and NOX-4 protein levels were higher in cortical and hippocampal microvessels as compared to cerebellum microvessels

**Fig 2:**
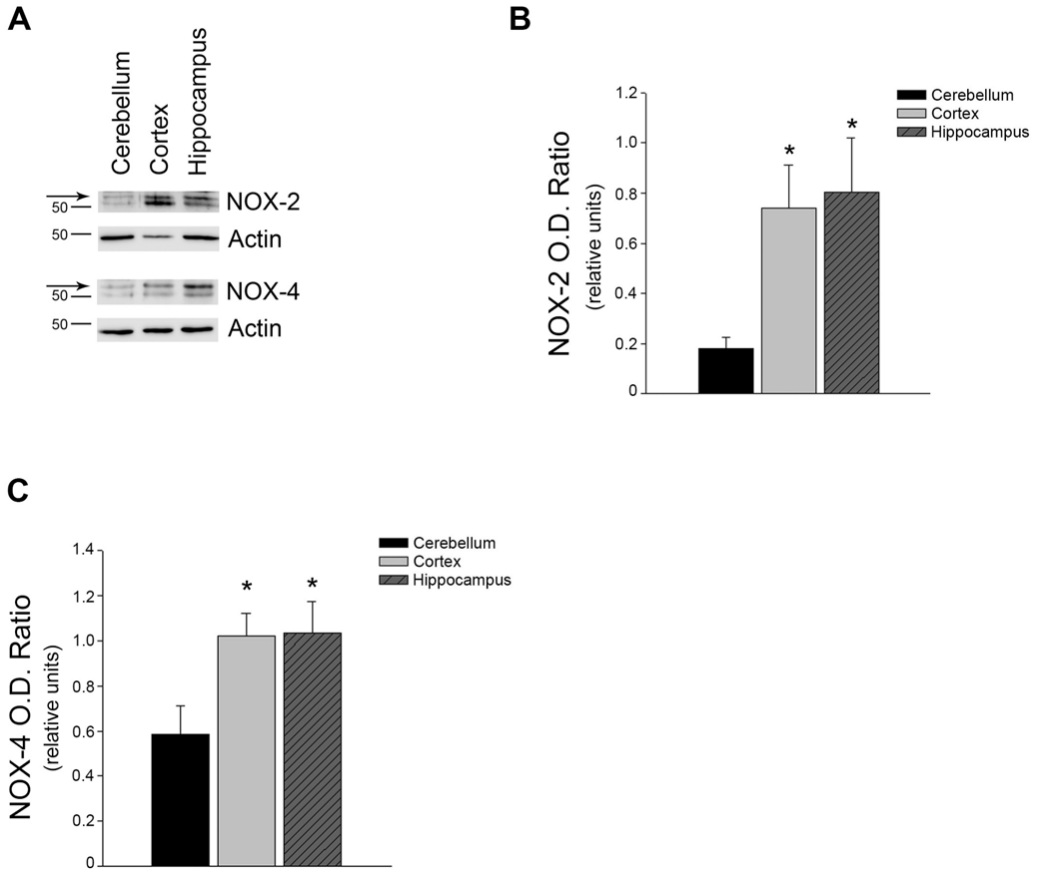
Protein levels of NOX-2 and NOX-4 were significantly increased in cortex and hippocampus microvessels. (A) Brain region microvessels were isolated, homogenized, and analyzed via Western blot analyses. Membranes were probed with anti-NOX-2 (n = 7) and anti-NOX-4 (n = 7–8) antibodies. A representative image is shown. (B) Densitometric analysis of NOX-2 and (C) NOX-4 levels were perfomed and normalized against Actin as a loading control. Data are presented as mean ± SEM (P<0.05).

NADPH oxidase (NOX) isoforms are the primary source of superoxide anions in the endothelium [18,19,20]. These enzymes function to produce superoxide anion while other sources of superoxide anion are simply a by-product or a product produced when proper enzyme activity is impaired [21,22]. To begin to examine the mechanism responsible for increased superoxide anion, we measured protein levels of NOX2 and NOX4, major NOX isoforms within the endothelium. Both NOX-2 and NOX-4 protein levels were significantly higher in the microvessels isolated from the cortex and hippocampus as compared to the cerebellum (Fig 2).

### MnSOD levels were lower in microvessels from the cortex and hippocampus

**Fig 3:**
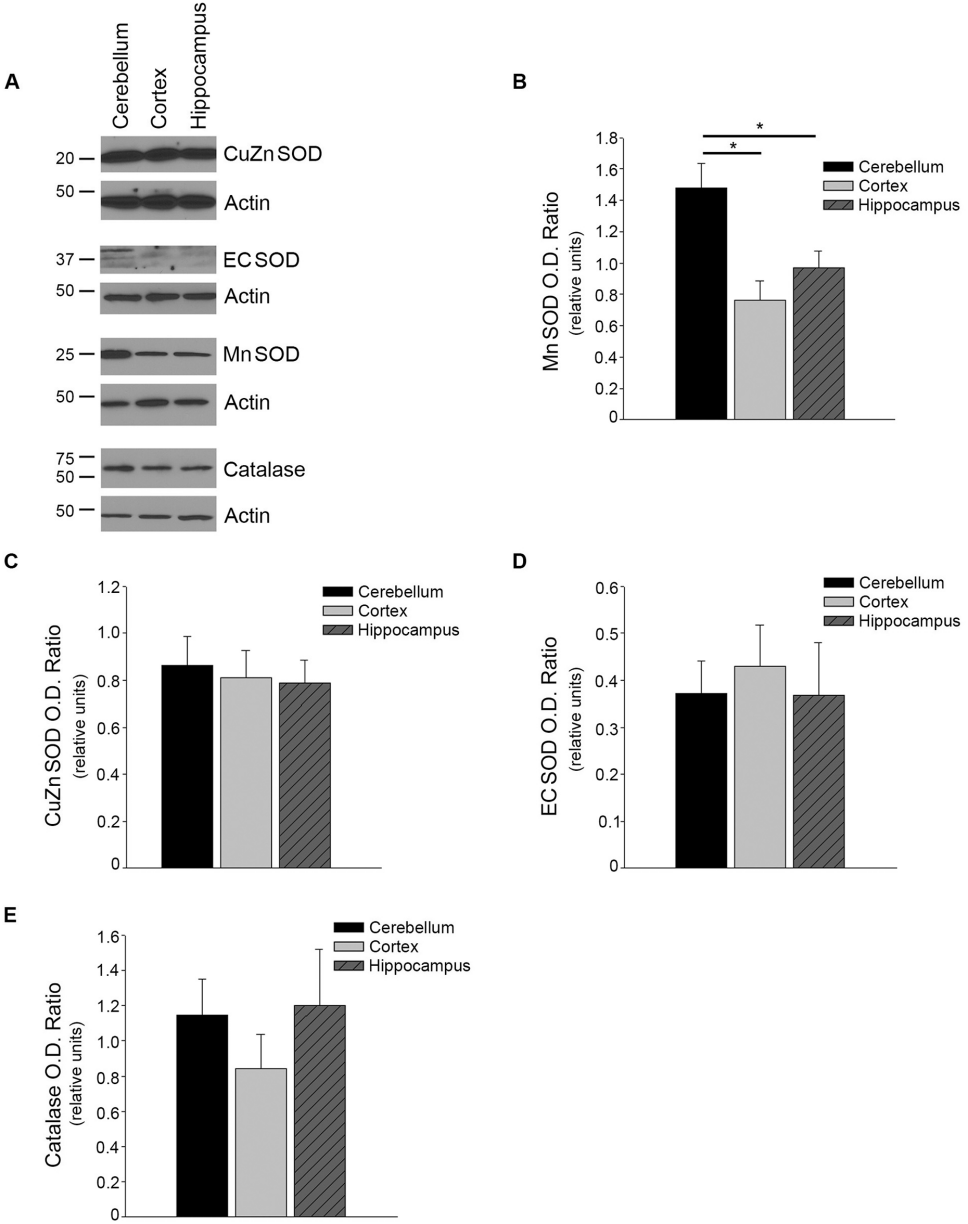
Levels of the antioxidant enzyme, MnSOD are decreased in the cortical and hippocampal microvessels. (A) Brain region microvessels were isolated and tissue homogenates were analyzed by Western blot analyses using anti- CuZnSOD (n = 9), ECSOD (n = 9), MnSOD (n = 10), and catalase (n = 4) antibodies. A representative image is shown. Densitometry analyses were performed normalizing against the loading control, Actin, for (B) MnSOD, (C) CuZnSOD, (D) ECSOD, and (E) catalase. Data are presented as mean ± SEM (P<0.05 as compared to cerebellum microvessels).

Antioxidant enzymes protect against cytotoxic effects of superoxide anion. We therefore examined protein levels of the major antioxidant enzymes in endothelial cells. Protein levels of several antioxidant enzymes were measured. MnSOD protein expression levels were significantly lower in the microvessels isolated from the cortex and hippocampus as compared to microvessels isolated from the cerebellum (Fig 3A and 3B; n = 10, P<0.05). Levels of CuZnSOD, ECSOD, and catalase were similar between the three regions (Fig 3C–3E, P>0.05).

### Tetrahydrobiopterin bioavailability was decreased in cortical and hippocampal microvessels

**Fig 4:**
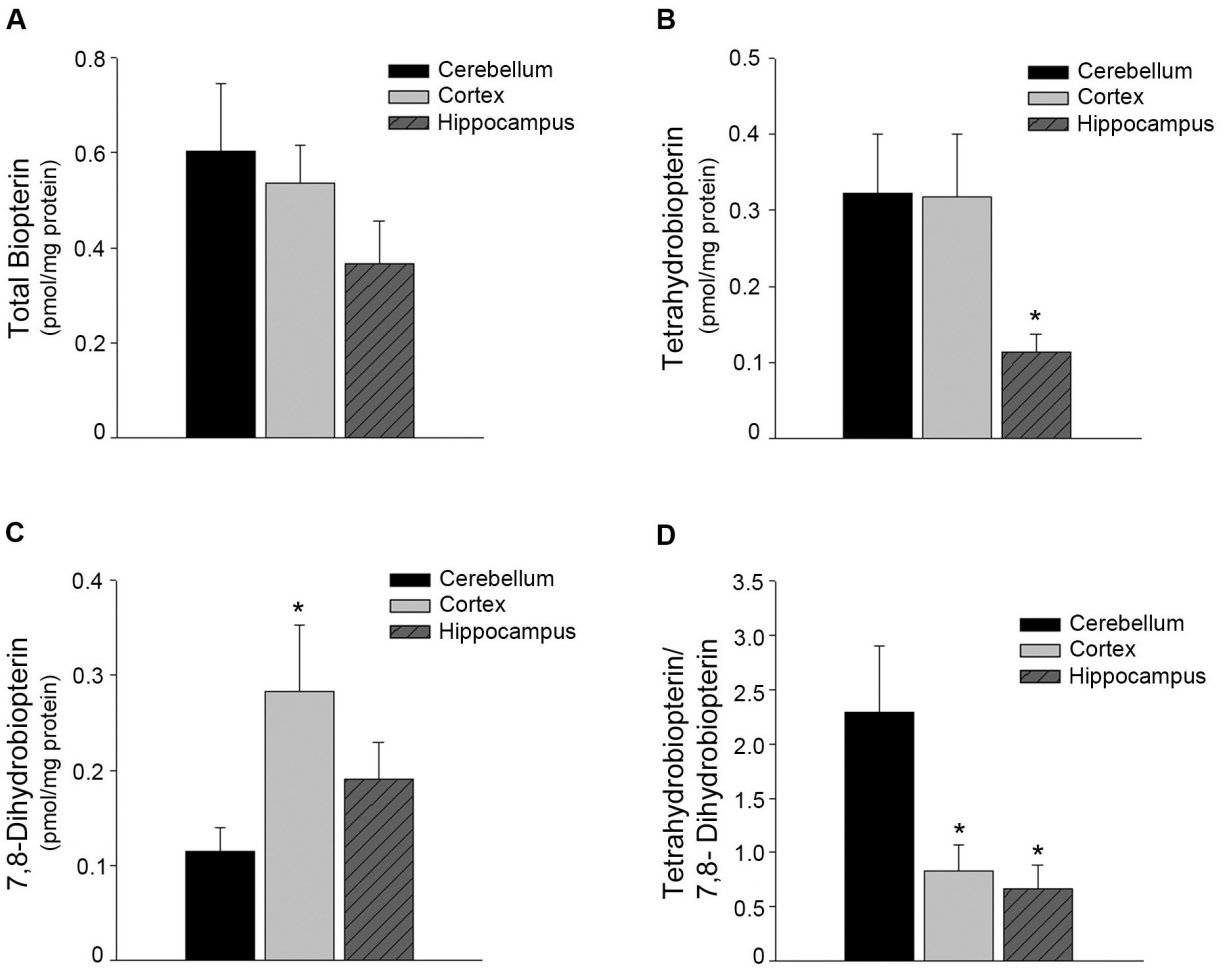
BH_4_ bioavailability was decreased in cortical and hippocampal microvessels as compared to microvessels from the cerebellum. Biopterin levels, BH_4_ and BH_2_, were analyzed by HPLC. (A) Total biopterin levels were unchanged between microvessels isolated from the three brain regions. (B) BH_4_ levels were significantly lower in the hippocampal microvessels as compared to the cerebellum. (C) BH_2_, the oxidized product of BH_4_, was significantly higher in the cortical microvessels as compared to the cerebellum. (D) The ratio of BH_4_:BH_2_, indicative of the bioavailable levels of BH_4_, is significantly lower in both the cortical and hippocampal microvessels as compared to the cerebellum. Data is presented as mean ± SEM (n = 7–9, P<0.05).

Tetrahydrobiopterin, an essential cofactor for proper eNOS enzyme activity and production of nitric oxide (NO), is a molecular target for oxidative stress. Total biopterin levels were not different between microvessels isolated from the cerebellum, cortex, and hippocampus (Fig 4A; n = 7–9, P>0.05). However, BH_4_ levels were lower in hippocampal microvessels as compared to cerebellum and cortical microvessels (Fig 4B; n = 7–9, P<0.05). Levels of the oxidized product, 7,8-dihydrobiopterin (BH_2_) were increased in the microvessels from the cortex as compared to the cerebellum (Fig 4C, n = 7–9, P<0.05). Hippocampal microvessels tended to have increased levels of BH_2_ as compared to the cerebellum but it did not reach statistical significance. Most notably, the ratio of BH_4_ to BH_2_, indicative of the bioavailable BH_4_, was lower in both cortical and hippocampal microvessels as compared to microvessels from the cerebellum (Fig 4D, n = 7–9, P<0.05).

### NOS uncoupling and cGMP levels

**Fig 5:**
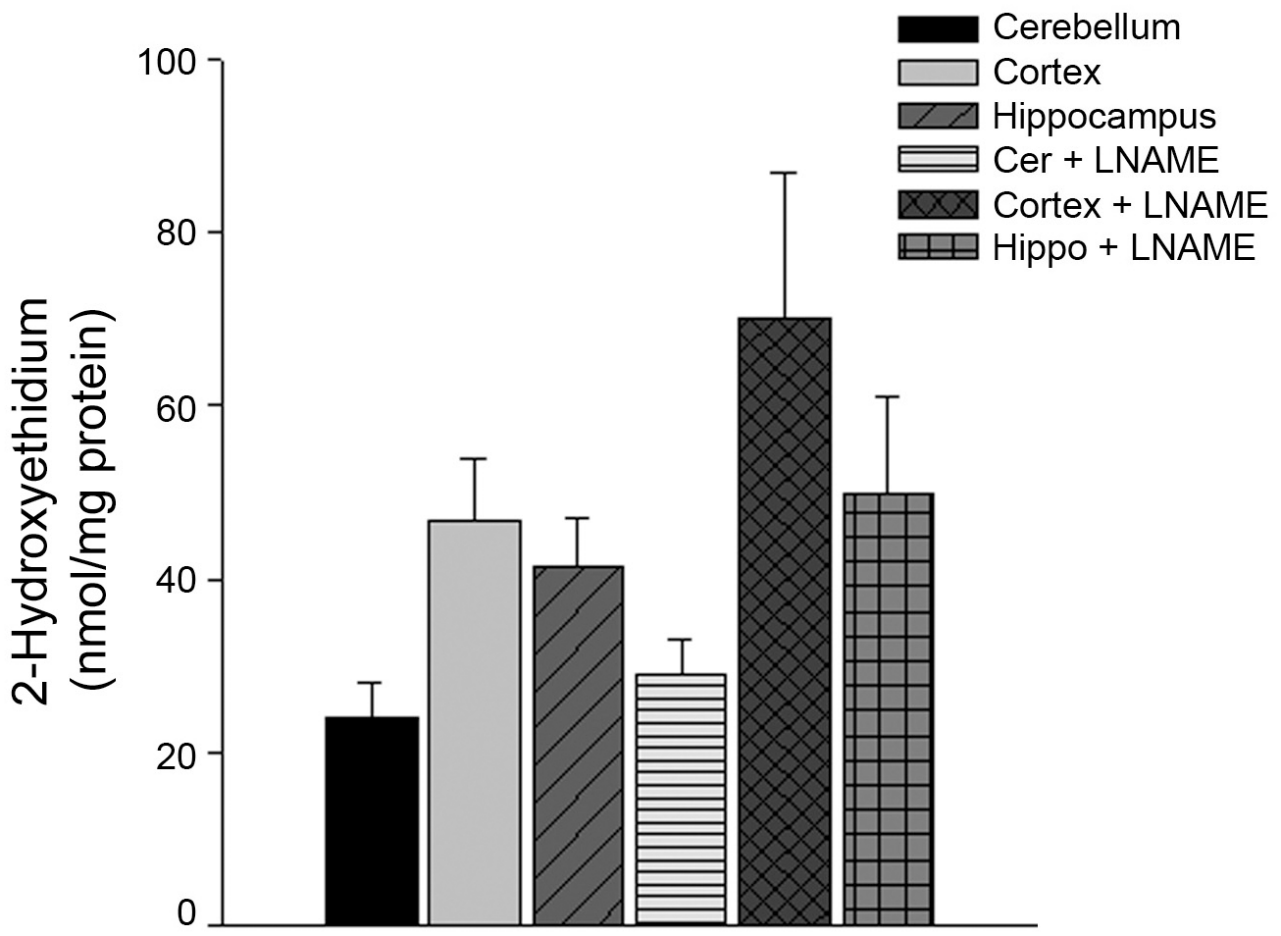
Pre-treatment with L-NAME, to inhibit NOS activity, did not significantly alter superoxide anion production. Regional microvessels were isolated and incubated with or without 30 μmol/L of L-NAME for 30 minutes at 37°. Following this incubation, microvessels were incubated with 50 μmol/L dihydroethidium at 37° for 15 minutes. Microvessels were homogenized in methanol and intracellular superoxide anions quantified by HPLC-based fluorescence. Intracellular superoxide anions were normalized to mg protein of each sample (n = 6). Data are represented as mean ± SEM.

**Fig 6:**
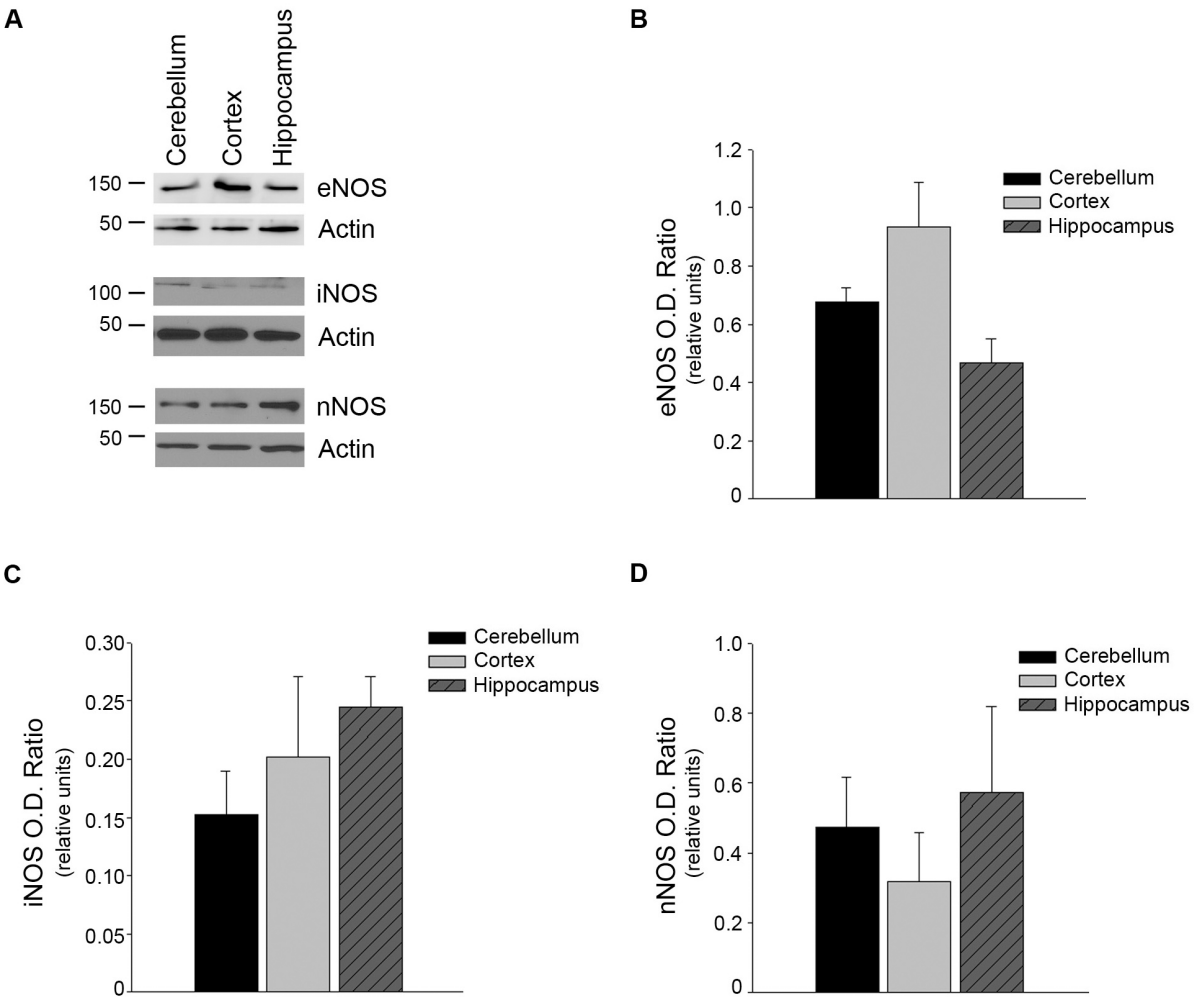
NOS protein levels were not altered between the microvessels of the cerebellum, cortex, or hippocampus. (A) Isolated brain region microvessels were homogenized and analyzed by Western blot analyses using anti- eNOS (n = 11), iNOS (n = 4), and nNOS (n = 5) antibodies. A representative image is shown. Densitometry analyses were performed normalizing against Actin, a loading control, for (B) eNOS, (C) iNOS, and (D) nNOS. Data are presented as mean ± SEM.

**Fig 7:**
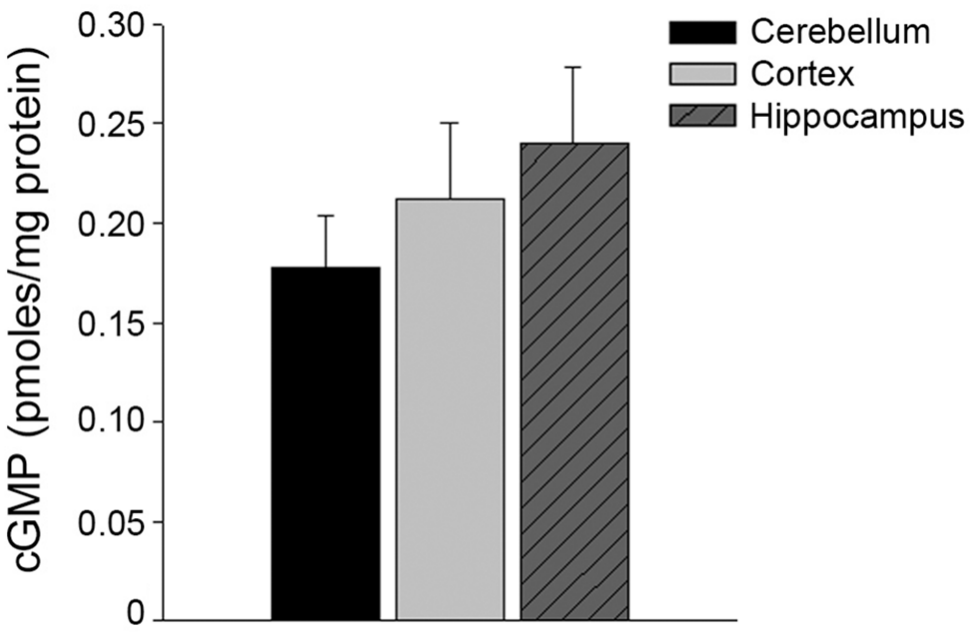
cGMP levels were not different between microvessels isolated from the cerebellum, cortex, or hippocampus. Regional microvessels were isolated and cGMP analyzed using a commercially available immunoassay.

eNOS uncoupling may also contribute to the generation of intracellular superoxide anions. To determine if superoxide anion production was caused by uncoupled eNOS, we measured superoxide in microvessels form the three regions, in the presence or absence of L-NAME. Treatment with L-NAME had no significant effect on superoxide levels in any of the three regions (Fig 5, n = 6, P>0.05). Furthermore, there was no difference in the levels of expression of any of the three NOS isoforms in the brain region microvessels (Fig 6A–6D, P>0.05). Lastly, cGMP, the major 2^nd^ messenger of NO, levels were similar between the cerebellum, cortical, and hippocampal microvessels (Fig 7, n = 8–9, P>0.05).

**Fig 8:**
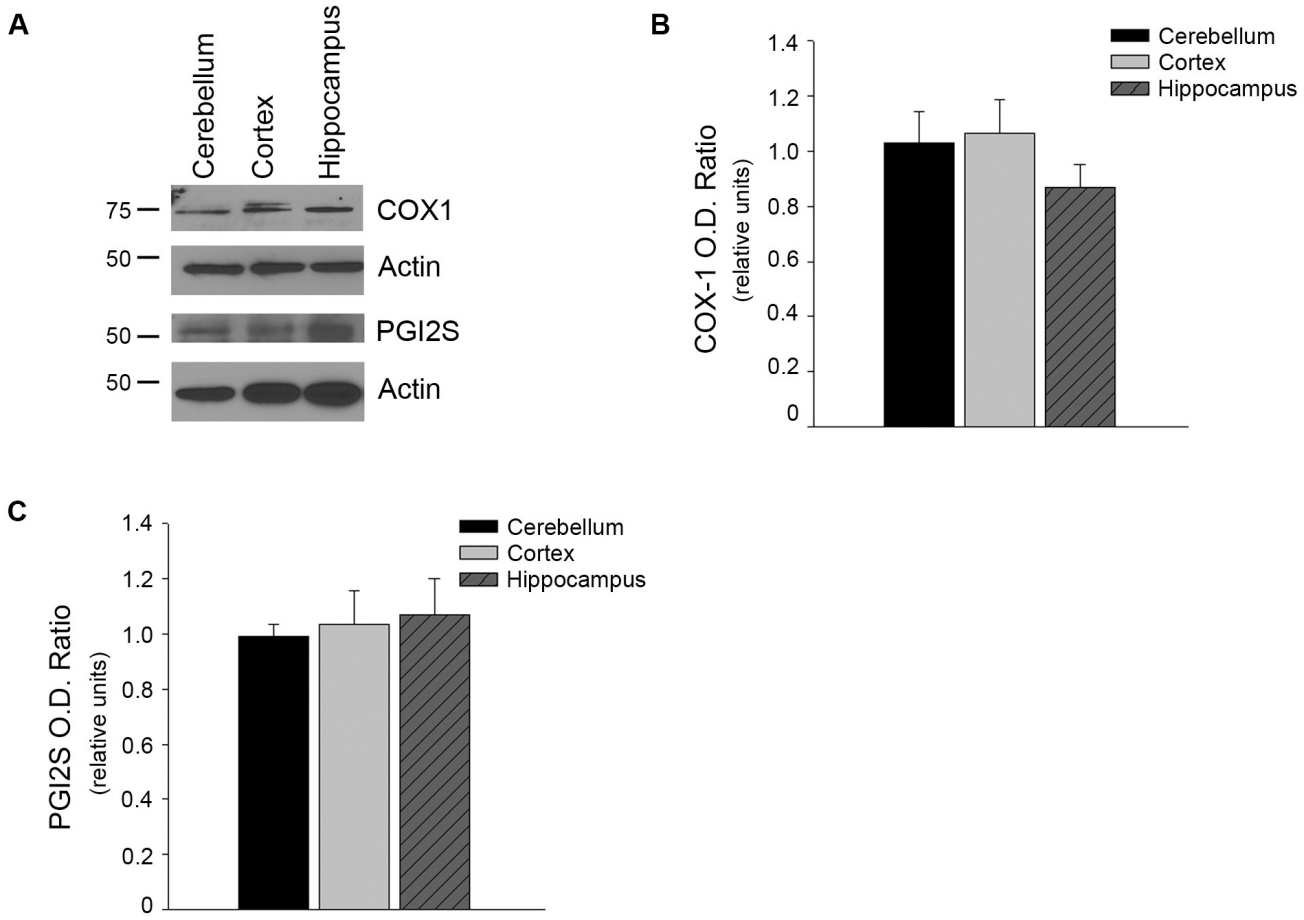
Protein levels of COX-1 and PGI-2S did not differ between brain region microvessels. (A) Isolated brain region microvessels were homogenized and analyzed by Western blot analyses using anti- COX-1 (n = 11) and PGI2S (n = 8) antibodies. A representative image is shown. Densitometry analyses were performed normalizing against Actin, a loading control, for (B) COX-1 and (C) PGI2S. Data are presented as mean ± SEM.

PGI_2_ is also an important vasoactive molecule within the vascular system having many similar functions as NO. However, we did not detect any significant changes in protein levels of PGI_2_ synthase or COX-1 (Fig 8).

## Discussion

We describe here several novel findings that may help elucidate cerebrovascular regional susceptibility to oxidative stress and other insults. First, we report that microvessels isolated from the cortex and hippocampus have significantly higher levels of intracellular superoxide anion. Second, we report increased protein levels of both NOX-2 and NOX-4. Third, protein levels of the antioxidant enzyme Mn SOD are significantly lower in these regions. Lastly, the bioavailability of BH4, an essential cofactor for proper eNOS enzyme activity, is significantly lower in cortical and hippocampal microvessels as compared to the cerebellum microvessels.

Superoxide anions contribute to cellular signaling; however, excessively high levels are a major contributor to the formation of reactive oxygen species which are quite toxic to cells/tissues. NOX is considered a primary source of superoxide in endothelial cells [18,19]. Both NOX2 and NOX4 were significantly increased in microvessels derived from the cortex and hippocampus as compared to the cerebellum and thus represent the most likely mechanism of increased superoxide anions observed in these regions. Our data suggests that eNOS is not uncoupled and therefore is not the source of superoxide anions. In addition, decreased antioxidant capacity might contribute to the elevation of intracellular superoxide anion concentrations. Indeed, we report here lower MnSOD protein levels in both the cortical and hippocampal microvessels which may contribute as a secondary mechanism to the higher intracellular superoxide anions within these microvessels.

Interestingly, bioavailability of BH_4_ is decreased in microvessels from the cortex and hippocampus as compared to the cerebellum. The oxidized product of BH_4_, BH_2_, is significantly higher in the cortical microvessels. It also tended to be higher in the hippocampal microvessels. Higher levels of BH_2_ and a decreased BH_4_:BH_2_ ratio are consistent with oxidation of BH_4_ by a higher concentration of reactive oxygen species. The hippocampal microvessels also had significantly lower levels of BH_4_ as compared to the other regions making it even more susceptible to oxidative stress. However, NOS protein levels are unaltered between the brain regions. Experiments using L-NAME, to inhibit NOS activity, suggest that NOS isoforms are not uncoupled in these microvessels. This conclusion is consistent with normal levels of cGMP in microvessels derived from the cerebellum, cortex, and hippocampus. Taken together, these data suggest that at this time eNOS activity is not altered by the decrease in BH_4_. Importantly, lower BH_4_ bioavailabilities make NOS highly susceptible to uncoupling in situations where oxidative stress may occur [23].

Vascular dysfunction may contribute to aging-dependent cognitive decline and AD. Oxidative stress and endothelium dependent vascular dysfunction are seen in aged vessels [24,25,26,27] as well as in AD [28,29,30,31]. We speculate that these regional differences may become exacerbated with age as the brain is exposed to oxidative insults. We do not know the functional importance or consequence of these physiological differences between the cerebellum and cortical and hippocampal microvessels; however, it is important to note that these variations may predispose the cortex and hippocampus microvessels to injury induced by oxidative stress thereby promoting endothelial dysfunction and increased vulnerability of surrounding neuronal tissue.

## Supporting Information

S1 Table Superoxide anion levels in brain region microvessels. (PDF)

S2 Table O.D. ratio values of NOX-2 and NOX-4 protein levels in brain region microvessels. (PDF)

S3 Table O.D. ratio values of antioxidants in brain region microvessels. (PDF)

S4 Table Biopterin levels in brain region microvessels. (PDF)
