# Supplementary material for: Regional Heterogeneity of Cerebral Microvessels and Brain Susceptibility to Oxidative Stress
Source: PLoS One. 2015 Dec 2;10(12):e0144062. doi: 10.1371/journal.pone.0144062 (PMC4668095; doi:10.1371/journal.pone.0144062)
Supplement: S1 Table — (PDF) [file pone.0144062.s001.pdf]

Superoxide values

	Cerebellum	Cortex	Hippocampus
1	11.68979	9.593684	5.763994
2	8.267975	12.37533	11.37108
3	3.091514	50.06292	36.90357
4	7.506694	32.5171	20.56401
5	3.738242	9.907715	9.56986
6	7.083262	18.93406	8.966933
7	7.69034	53.25187	54.73543
8	1.416769	5.015189	7.782432
9	2.036002	4.390629	5.617856
10	6.45388	6.882942	11.2761
11	3.166015	12.04694	5.410402
12	1.09808	3.074022	5.033791
13	6.374621	11.84001	3.624065
Average	5.35486	17.68403	14.35535
SEM	0.881099	4.68386	4.178138
