# Supplementary material for: Regional Heterogeneity of Cerebral Microvessels and Brain Susceptibility to Oxidative Stress
Source: PLoS One. 2015 Dec 2;10(12):e0144062. doi: 10.1371/journal.pone.0144062 (PMC4668095; doi:10.1371/journal.pone.0144062)
Supplement: S2 Table — (PDF) [file pone.0144062.s002.pdf]

Relative O.D. Ratios for NOX-2 and Nox-4

	NOX2				NOX4	
	Cerebellum	Cortex	Hippocampus		Cerebellum	Cortex
1	0.14541432	0.666859	1.3972973		1.115065653	1.199374
2	0.411877395	1.337725	0.5080549		0.31977321	1.295176
3	0.242452683	0.483668	0.5218072		1.10184021	0.976404
4	0.114112751	1.443128	0.5109894		0.337096373	0.739437
5	0.085597549	0.380544	0.588332		0.261240508	1.349978
6	0.157341472	0.430575	1.8196566		0.31360769	0.769606
7	0.11746072	0.429038	0.2811083		0.669245431	0.805253
8					0.564297479	
Average	0.182036698	0.738791	0.8038923	Average	0.585270819	1.019318
SEM	0.042681578	0.172127	0.215835	SEM	0.124347881	0.098345

Hippocampus
1.375092771
0.572166724
1.643080125
1.302479887
0.696680685
1.185982276
0.850482084
0.635842194
1.032725843
0.140265864
