# Supplementary material for: Regional Heterogeneity of Cerebral Microvessels and Brain Susceptibility to Oxidative Stress
Source: PLoS One. 2015 Dec 2;10(12):e0144062. doi: 10.1371/journal.pone.0144062 (PMC4668095; doi:10.1371/journal.pone.0144062)
Supplement: S3 Table — (PDF) [file pone.0144062.s003.pdf]

Relative O.D. Ratios- antioxidants

MnSOD

	Cerebellum	Cortex	Hippocampus
1	1.4551928	1.20072	1.103336
2	2.6342129	1.398923	1.669861
3	1.9214502	1.091163	1.424911
4	1.5856821	0.350216	0.837073
5	1.352374	0.653089	0.621944
6	0.9887464	0.920218	1.007851
7	0.9748974	0.254731	0.584577
8	1.1232323	0.891574	0.824726
9	1.5492081	0.335327	0.815803
10	1.1822494	0.532401	0.795544
Average	1.4767246	0.762836	0.968563
SEM	0.1588933	0.12569	0.109474

CuZn SOD

	Cerebellum	Cortex	Hippocamp
1	1.04767425	0.929425	0.88571
2	0.56873402	0.980159	1.239908
3	0.8801628	0.687605	0.889726
4	0.15278939	0.067105	0.605524
5	0.69262749	0.470786	0.919832
6	1.30178144	1.176964	0.971831
7	1.06875989	1.066427	0.196735
8	1.29752511	0.843897	0.769723
9	0.76372488	1.062111	0.617953
Average	0.86375325	0.809387	0.788549
SEM	0.12292423	0.117277	0.097712

EC SOD

	Cerebellum	Cortex	Hippocampus
1	0.3478261	0.350819	0.309537
2	0.2874585	0.194712	0.072082
3	0.0295066	0.739448	0.143181
4	0.1761364	0.812282	0.007957
5	0.6308163	0.410468	0.755869
6	0.7062188	0.714419	0.966796
7	0.4325681	0.301445	0.378319
8	0.3936192	0.22929	0.585478
9	0.3450226	0.120587	0.091998
Average	0.3721303	0.430386	0.367913
SEM	0.069417	0.08639	0.112035

Catalase

	Cerebellum	Cortex	Hippocamp
1	1.25659691	0.511544	2.132612
2	1.63196924	1.093495	0.858051
3	0.68057121	1.251463	0.738781
4	1.01783355	0.511145	1.077081
Average	1.14674273	0.841912	1.201632
SEM	0.20030106	0.193558	0.318135

ous

ous
