# Supplementary material for: Regional Heterogeneity of Cerebral Microvessels and Brain Susceptibility to Oxidative Stress
Source: PLoS One. 2015 Dec 2;10(12):e0144062. doi: 10.1371/journal.pone.0144062 (PMC4668095; doi:10.1371/journal.pone.0144062)
Supplement: S4 Table — (PDF) [file pone.0144062.s004.pdf]

HPLC- Biopterin levels

Total Biopterin

	Cerebellum	Cortex	Hippocampus
1	0.741536653	0.658375941	0.310585307
2	0.586684247	0.192329143	0.268328275
3	1.28655584	0.753175959	0.223353738
4	0.131601947	0.693637131	0.167228274
5	0.633545158	0.666108119	1.01532112
6	1.192762699	0.363234751	0.5705848
7	0.480055269	0.42263536	0.286783945
8	0.167792613		0.213296119
9	0.207780515		0.247430764
Average	0.603146105	0.535642343	0.36699026
SEM	0.140356465	0.079391584	0.089720636

BH4

	Cerebellum	Cortex
1	0.63694261	0.539629726
2	0.376629468	0.048863436
3	0.093398627	0.346534034
4	0.406297884	0.154850632
5	0.594523687	0.191164083
6	0.361666995	0.769886304
7	0.070907836	0.238806509
8	0.122112361	0.248540678
9		
Average	0.332809934	0.317284425
SEM	0.077856104	0.082415197

BH2

	Cerebellum	Cortex	Hippocampus
1	0.104594043	0.118746216	0.186231035
2	0.210054779	0.143465707	0.065762354
3	0.034715119	0.406641924	0.143464038
4	0.038203321	0.538786499	0.104594468
5	0.227247274	0.474944036	0.316857895
6	0.118388274	0.124428241	0.323938908
7	0.096884777	0.174094683	0.248384327
8	0.085668154		0.117896586
9			0.213535847
Average	0.114469468	0.283015329	0.191185051
SEM	0.025101325	0.069180761	0.030752232

Ratio

	Cerebellum	Cortex
1	6.089664288	0.340593142
2	1.793005944	0.85218472
3	2.444777699	0.287406295
4	1.787910923	0.402498123
5	0.993789565	0.585160729
6	3.054922444	1.919230769
7	0.731877987	1.427617856
8	1.425411376	
9		
Average	2.290170028	0.830670233
SEM	0.60357605	0.235062854

Hippocampus
0.124354272
0.202565921
0.0798897
0.062633806
0.246645892
0.038399618
0.135583589
0.095399533
0.033894917
0.113263028
0.024234745
Hippocampus
0.667741935
0.556862203
0.59882522
2.204342187
0.761396317
0.154597588
0.090388605
0.809179771
0.158731741
0.666896174
0.212873818
